# Managing Alcohol Use Disorder in Primary Health Care

**DOI:** 10.1007/s11920-017-0837-z

**Published:** 2017-09-14

**Authors:** Peter Anderson, Amy O’Donnell, Eileen Kaner

**Affiliations:** 1 Institute of Health & Society, Newcastle University, Baddiley-Clark Building, Richardson Road, Newcastle upon Tyne, NE2 4AX UK; 2 Faculty of Health, Medicine and Life Sciences, Maastricht University, P. Debyeplein 1, 6221 HA Maastricht, Netherlands

**Keywords:** Heavy drinking, Alcohol use disorder, Primary health care, Screening, Brief advice, Implementation, Community

## Abstract

**Purpose of Review:**

The aim of this study is to summarise the current literature on both the impact and the implementation of primary health care-based screening and advice programmes to reduce heavy drinking, as an evidence-based component of managing alcohol use disorder in primary health care.

**Recent Findings:**

Systematic reviews of reviews find conclusive evidence for the impact of primary health care delivered screening and brief advice programmes in reducing heavy drinking. The content, length of advice and which profession delivers the advice seems less important than the actual encounter between provider and patient. Despite the global burden of disease due to heavy drinking and the evidence that this can be reduced by screening and brief advice programmes delivered in primary health care, such programmes remain poorly implemented. Were such programmes widely implemented, there would be substantial health and productivity gains. Systematic reviews and international studies indicate that improved implementation requires tailoring of training and programme content to match the needs of providers, training and ongoing support and embedding of programmes within local community support, championed by local leaders.

**Summary:**

The next stage of implementation and scale-up of evidence-based screening and brief advice programmes should take place embedded within supportive local community action, with appropriate research to demonstrate impact.

## Introduction

Heavy drinking is a cause of ill-health and premature death [1]. Even though heavy drinking can be prevented and managed, the World Health Organisation (WHO) has estimated that as many as four out of five heavy drinking individuals do not receive an offer of evidence-based advice and treatment [2, 3]. As a lifestyle risk factor, alcohol consumption is a leading cause of global ill-health and premature death, making action to prevent and treat heavy drinking a priority for policy and practice. In this overview, we critically evaluate recent literature on primary health care-based interventions for managing heavy drinking. We begin with a brief summary of the epidemiology of heavy drinking, including an examination of the relevancy and utility of the various definitions of problem drinking currently in use. We move on to summarise the findings of latest research on interventions for heavy drinking, with a specific focus on screening and brief alcohol advice. We conclude with some observations on the current challenges faced by the field, including thoughts on how the slow rate of implementation might be addressed.

## Epidemiology of Alcohol Use Disorder

Alcohol is a cause of a wide range of diseases and injuries, exacerbated by occasions of heavy drinking [4•], resulting in it ranking as the ninth leading global risk factor for morbidity and premature death [5•]. There are more than 40 ICD-10 three-digit disease categories that are fully attributable to alcohol, including neuro-psychiatric disorders, intoxication and dependence, gastrointestinal diseases, poisoning and foetal alcohol syndrome, as part of foetal alcohol spectrum disorders. Alcohol is a carcinogen, being a cause of cancers of the oral cavity, larynx, pharynx, oesophagus, colon and rectum, liver and breast [6, 7]. Most partially attributable disease categories show dose-response relationships with the volume of alcohol consumed: the higher the level of consumption, the higher the risk of ill-health or premature death. Exceptions are ischaemic heart disease and diabetes, which show curvilinear relationships, with, compared to abstainers, lower risks at low doses of consumption, and higher risks at high doses of consumption. The absolute number of alcohol-attributable DALYs (disability adjusted life years, a summary measure of ill-health and premature death) increased by about 25% between 1990 and 2015, largely driven by changes in population growth, population ageing, and background rates of diseases for which alcohol is an attributable cause. The adverse impacts from heavy drinking are exacerbated by lower income. For any given level of alcohol consumption, poorer people suffer more harm than richer people [8]. Harm also occurs to people other than the drinker, with considerable harms extending to families, communities, health systems and economies as a whole [9].

The bulk of severe alcohol-related health problems, including mortality, occurs in middle age [10] and, it is amongst this age group that policy and programme interventions are likely to bring the greatest health and productivity gains [11•]. Heavy drinkers are responsible for the majority all alcohol-related harm [12]. It is also amongst this group, compared with lighter drinkers, that disproportionally greater health gains can be made for the same absolute reduction in alcohol consumption [13]. Thus, if advice and treatment programmes are to be most efficient in reducing the harm done by alcohol, they should preferentially address adult drinkers, and, in particular, those who drink heavily.

### Alcohol Use Disorder

Alcohol use disorder (AUD) is a summary term used for the two diagnosable conditions of “harmful use of alcohol” and “alcohol dependence” within the WHO ICD-10 classification of mental and behavioural disorders [14]. AUD will also be included in the ICD-11 revision under disorders due to the use of alcohol [15]. Similarly, AUD is a diagnosable condition within the 5th edition of the Diagnostic and Statistical Manual of Mental Disorders (DSM–5) of the American Psychiatric Association [16]. The most recent global burden of disease analyses estimate that globally, there were between 63.5 [17] and 95 million cases [18] of AUD in 2015, leading to 137,500 deaths [19], 6.3 million years lived with disability [17] and 112 million disability-adjusted life years [20].

### Harmful Use of Alcohol

WHO also uses the term the harmful use of alcohol [14]. As a clinical term, it is a pattern of alcohol use that is causing damage to health. This designation will be replaced in 2017 with two clinical terms: *a harmful pattern of alcohol use* (a pattern of alcohol use sustained over at least 12 months that has clinically significantly harmed the health of the user or someone else); and, *a single episode of harmful use of alcohol that has caused damage to a user’s health or someone else’s health* [15]. Harmful use of alcohol is a term used (albeit, with different definitions) in the global non-communicable disease framework [21], the WHO global strategy to reduce the harmful use of alcohol [22], and the United Nations sustainable development goals [23].

### United Nations Sustainable Development Goals

In 2015, United Nations launched the sustainable development goals (SDGs) (http://www.un.org/sustainabledevelopment/sustainable-development-goals/) for the 15-year period 2016–2030. For good health and well-being, Goal 3, Target 3.5 is to strengthen the prevention and treatment of substance abuse, including narcotic drug abuse and harmful use of alcohol [23]. The two key indicators are: 3.5.1, coverage of treatment interventions (pharmacological, psychosocial and rehabilitation and aftercare services) for substance use disorders (including alcohol use disorders); and, 3.5.2, harmful use of alcohol, defined according to the national context as alcohol per capita consumption (aged 15 years and older) within a calendar year in litres of pure alcohol.

### Summary Exposure Value

A summary exposure value (SEV) has been proposed by the Global Burden of Disease studies as an indicator to monitor achievement of health-related SDGs. For alcohol, SEV is estimated from a combination of average daily alcohol consumption of pure alcohol (measured in g/day) in current drinkers who had consumed alcohol during the past 12 months and the proportion of the population reporting heavy episodic drinking, defined as consumption of at least 60 g for males and 48 g for females of pure alcohol on a single occasion [5•]. SEV ranges from 0% (no risk exposure in a population) to 100% (entire population has maximum possible risk). The global age-standardised SEV for alcohol remained stable for men between 1990 (SEV = 10.9%) and 2015 (SEV = 10.7%), whereas it decreased for women from 5.9% in 1990 to 5.1% in 2015, with most of the decrease occurring in the years 1990 to 2005.

Structural drivers of alcohol exposure are socio-demographic changes, which have been aggregated as an index (SDI) based on estimates of lag-dependent income per capita, average educational attainment over the age of 15 years and total fertility rate, scaled from zero to one. Globally, as SDI goes up, alcohol exposure goes up [5•]. The exception to this is within higher income countries (which have overall higher alcohol exposure than lower income countries), where, within country groupings, there is a tendency for countries with higher SDIs to have lower alcohol exposure.

### Focus on Heavy Drinking

Alcohol use disorder and harmful use of alcohol are not easy or simple to understand concepts when screening for risky patients in primary health care [24, 25]. An easier concept to understand is heavy drinking based on a level of alcohol consumption [26, 27]. The European Medicines Agency [28] provides an operational definition of heavy drinking: ‘threshold 1’ defines heavy drinking as more than 60 g of alcohol consumed on average a day by a man and more than 40 g a day by a woman. These levels are the same as those used in the original global burden of disease studies [29]. For operational purposes, the mid-point (50 g a day) can be taken as a definition of heavy drinking. Roughly speaking, 50 g of alcohol is contained in three and a half 12-oz (355 ml) cans of 5% beer, two thirds of a bottle of 12% wine (3–4 glasses, depending on the size poured) and three and a half shots of spirits (1.5 oz, 44 ml, of 40% alcohol). At this level of consumption, there is little difference in absolute risk between men and women of dying prematurely due to alcohol before the age of 70 years, where the risk is about 3.5% [30].

**Table:** 

With respect to alcohol use disorder and the harmful use of alcohol, we recommend the term heavy drinking for use in primary health care settings, as it is easier to understand, and is the focus of the vast majority of primary health care-based studies to date.	

## Impact of Screening and Brief Advice Delivered in Primary Health Care

Primary health care is the setting which can improve access to health care, particularly for the poor, at reasonably low cost in all countries, from low- to high-income. Primary health care is a vehicle to achieve universal health coverage, reduce inequities, and promote shared decision-making between providers and patients through participation, and collaborative care models throughout health systems [31]. Screening and brief advice to reduce heavy drinking in primary health care comprises two key elements: first, use of a short, but validated, questionnaire to help identify those individuals drinking heavily, with consistently good performance reported for the AUDIT-C instrument [32•]; and, second, delivery of brief advice and treatment, designed to promote awareness of the negative effects of heavy drinking and to motivate reduction in drinking, often based on the FRAMES principles [33].

### AUDIT-C

The three questions of the AUDIT-C assess different dimensions of alcohol consumption, with each question scored on a different scale (i.e. drinking days per week, drinks per drinking day and frequency of heavy drinking). Thus, the summed score does not necessarily reflect any one pattern of drinking, and increasing scores are not necessarily related to linear increases in any given dimension of consumption. Nevertheless, AUDIT-C scores are associated with several alcohol-related health risks, including alcohol dependence, severity of problem drinking, postoperative complications, hospitalizations for gastrointestinal conditions, trauma and mortality, generally in a dose-response manner [34]. AUDIT-C also captures the summary exposure value defined for alcohol, a composite of average consumption and heavy episodic drinking [5•].

### Brief Advice in Primary Health Care

Brief advice delivered in primary health care is commonly 5–10 min in duration and often based on the ‘FRAMES principles’ and the ‘Five As’ [33]. FRAMES is an acronym summarising the key components of brief advice: feedback (on the client’s risk of having alcohol problems); responsibility (change is the client’s responsibility); advice (provision of clear advice when requested); menu (what are the options for change?); empathy (an approach that is warm, reflective and understanding); and self-efficacy (optimism about the behaviour change). The five As are: (1) *assess* alcohol consumption with a brief screening tool, followed by clinical assessment as needed; (2) *advise* patients to reduce alcohol consumption to lower levels; (3) *agree* on individual goals for reducing alcohol use or abstinence (if indicated); (4) *assist* patients in acquiring the motivations, self-help skills or support needed for behaviour change; and, (5) *arrange* follow-up support and repeated counselling, including the referral of dependent drinkers to specialty treatment.

A series of systematic reviews over 15 years, covering a total of 56 unique primary health care-based randomised controlled trials, has consistently found that, up to 12-months follow-up, commonly the longest period studied, brief advice is effective in reducing heavy drinking, leading to lower average alcohol consumption, a reduction in alcohol-related problems, and reduced health care utilisation and mortality outcomes [35••].

Delivery by a range of practitioners has beneficial effects, and there is little evidence to suggest that any one profession of provider performs better or worse than another [36••]. Further, there is little evidence to suggest that the content of the advice is important for the outcome, or that longer or more sophisticated advice leads to better outcomes than shorter or less sophisticated advice [36••]. So, it seems that the length, complexity and sophistication of the advice are less important than the actual contact between provider and patient. Further, two systematic reviews that studied outcomes amongst control groups in studies of brief advice [37, 38] found consistent evidence of reduced drinking. Thus, what is termed screening or assessment reactivity may be additional elements of the positive effects of brief advice.

Most of the evidence for brief advice has focused on adults aged between 18 and 65 years, rather than young or older people [35••]. Thus, it is not possible to conclude that brief advice works just as well for the young and elderly as it does for adults.

### Digital-based Advice

There has been considerable development both to supplement primary health care-based screening and brief advice programmes, and to extend the reach of screening and brief advice through digital-based interventions. A Cochrane systematic review that included 40 trials compared the drinking of people getting advice about alcohol from computers, telephones or internet sites against those that did not [39, 40]. Overall, participants randomised to a digital intervention drank on average 23.6 (95% CI 16.0, 31.2) grams of alcohol (roughly two drinks) per week less than controls, a proportional reduction similar in size to that achieved by face-to-face advice [35••]. The reduction appeared to be sustained across lengthening follow-up, although it was not statistically significant by 12 months. Of the 40 trials, 26 were solely of adolescents, young adults or college students. There was a statistically significant, although smaller, reduction in consumption in this population of 14.0 g per week (95% CI 8.1, 19.9). Five trials provided information on alcohol consumption by sex. There was no evidence from these trials that the difference in alcohol consumption between trial arms was modified by sex, but the available data were limited. Five trials reported a direct comparison between a digital and face-to-face intervention. There was no evidence from these trials of a difference in alcohol consumption between these arms. Four out of five studies investigating cost-effectiveness, reported the intervention was cost-effective. There was no evidence to suggest that the length of the intervention or the specific type of digital intervention impacted on cost-effectiveness. Of the behaviour change techniques, uniquely present in experimental arms, i.e. not present in both experimental and control arms, the five most frequently used were: ‘Feedback on behaviour’ (82.9%), ‘Social comparison’ (80.5%), ‘Information about social and environmental consequences’ (70.7%) ‘Feedback on outcomes of behaviour (65.9%) and ‘Social support (unspecified)’ (65.9%). There was not enough information to determine if advice was better from computers, telephones or the internet. Advice from trusted places like doctors’ groups (credible source) seemed helpful as well as suggestions about things to do instead of drinking (behaviour substitution).

### Other Settings

The impact of brief advice programmes in antenatal care has infrequently been studied and there is no conclusive evidence that suggests an impact of brief advice programmes delivered in antenatal care [41]. There is a building evidence base for the feasibility and effectiveness of delivering screening and brief advice for heavy drinking in pharmacy settings, although there is insufficient evidence to date to propose widespread roll out [42]. Compared with primary health care, there is a smaller evidence base for the impact of brief advice undertaken in emergency care settings [41]. One meta-analysis based on 33 publications covering 28 individual studies found that 6 out of 9 meta-analyses, comparing change from baseline score differences between brief advice and control conditions, presented significant results favouring brief advice. However, the effect sizes were small, with the highest standardised mean difference amounting to 0.19 (95% CI 0.08–0.31), suggesting a cautious approach to widespread roll out of brief advice programmes undertaken in emergency care settings [43].

**Table:** 

Screening and brief advice delivered in primary health care is effective in reducing heavy drinking. The evidence is stronger than in other settings. The actual content and length of the brief advice seems less important than the contact between provider and patient.	

## Implementing Screening and Brief Advice Programmes in Primary Health Care

### Managing Screening

AUDIT-C scores for triggering brief advice in primary health care are commonly set at five for both men and women, or five for men and four for women. These levels reflect a consumption of about 20 g of alcohol or less per day [34]. Primary health care providers may be unwilling to give advice at such low levels of consumption, more so because it would become very time consuming, with as many as one in three or four patients being eligible for advice.

In the case of managing hypertension, cut-off levels are normally taken as levels of blood pressure at which treatment has shown to be effective [44]. Similarly, cut-off levels for heavy drinking could be taken as levels of alcohol consumption found at which brief advice has been found to be effective. In the first Cochrane review of the topic, these levels were found to be, on average, 313 g per week [45••]. At a daily average of 45 g, the relevant AUDIT-C cut-off score is 8 [34]. That lower cut-offs may be a constraining factor is also illustrated by the lower effect sizes found in an update of the Cochrane review [46••] (half in size from the original Cochrane review [45••]), where the average baseline consumption at enrolment had dropped to 183 g/week (26 g/day).

It has also been proposed that primary health care providers might be more active in screening for heavy drinking, if screening were restricted to patients with comorbid conditions, such as high blood pressure or depression [47–49]. To date, though, there are no available evidence-based packages that deal with comorbidity to implement [50]. Further, such restrictive screening misses most screen-positive patients that are identified through universal screening [51].

### Evidence for Increasing Primary Health Care Activity

Two systematic reviews [52, 53•] and two multi-country studies [54•, 55•, 56] have provided evidence on how to increase the activity of primary health care providers in screening patients and in advising screen-positive patients. The WHO Phase III four-country study on the identification and management of alcohol-related problems in primary care found that the odds ratios for the impact of training and support on increased screening (defined as 20% or more of eligible patients screened) was 2.2 (95% CI = 1.3 to 3.1) and on increasing higher intervention (defined as 10% or more of eligible patients screened and advice given to screen positives) was 2.8 (95% CI = 1.6 to 4.0); albeit from very low baseline levels [56].

The five-country European ODHIN (optimising delivery of health care interventions) study tested the impact of training and support and of financial reimbursement in changing provider behaviour. Providers who had received training and support screened 50% more patients that providers who had not received training and support; providers who received financial reimbursement screened 100% more patients than providers who did not receive financial reimbursement [54•]. In both cases, the baseline levels of screening were low, at 6/100 consulting adult patients screened [54•]. In contrast, evidence from routine practice in England practice found limited effects of financial incentives on provider’s screening and advice behaviours [57].

The WHO Phase IV 12-country study on the identification and management of alcohol-related problems in primary care concluded that primary health care activity could be enhanced through: (i) local customization of training and practice-based materials; (ii) reframing views about alcohol of both professionals (through training) and the public (through mass media campaigns); (iii) establishment of a lead organisation with endorsements and support from a range of organisations and individuals to provide focused leadership; and (iv) adequately controlled community-based studies to strengthen the evidence base for achieving routine implementation [58••]. These actions place primary health care-based screening and brief advice programmes in the context of community and municipal environments, in which additional support can help improve outcomes. In the USA, the SBIRT (screening, brief intervention and referral to treatment) programme led by SAMHSA (Substance Abuse and Mental Health Services Administration) [59] identified that local champions and whole primary health care centre buy-in were needed for successful implementation [60, 61].

**Table:** 

The volume of screening and brief advice delivered in primary health care can be enhanced with training and support. It is likely, although not yet fully evaluated, that the volume could be further enhanced by embedding screening and brief advice within a frame of broader supportive community action.	

## Implementation Strategies

**Fig. 1:**
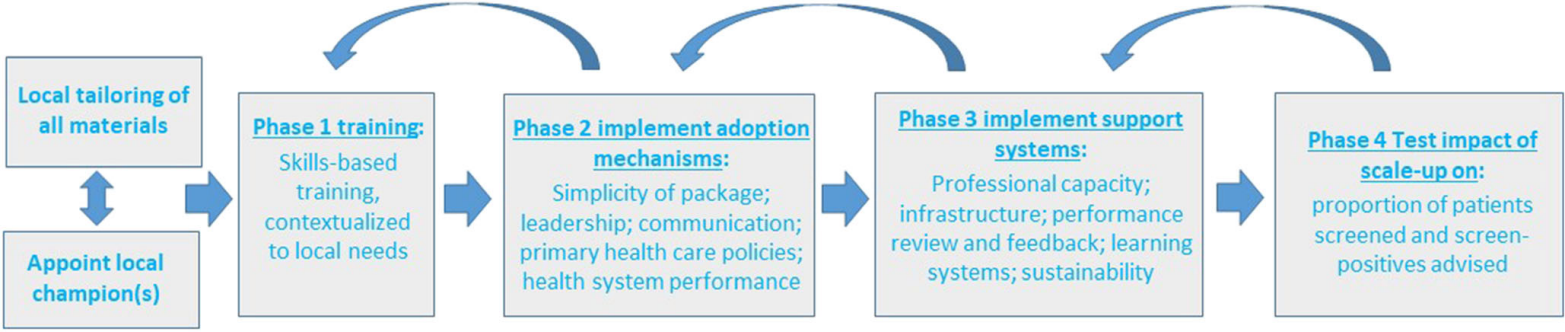
Phases for enhanced implementation of screening and brief advice programmes, embedded within community support

We view one of the main reasons for failing to achieve large-scale increases in PHC-based activity as due to implementation strategies focussing on health care providers alone, whereas successful implementation of health interventions within health systems requires managing relevant broader structural and support systems [62•]. In the final part of this review, we outline a number of mechanisms that could be used to embed primary health care activity within broader community support so as to enhance the volume of screening and brief advice delivered, Fig. 1.

### Local Champions

A champion, who acts as a trusted knowledge and practice broker can be identified and appointed with the responsibilities of promoting the implementation of screening and brief advice programme serving within communities [62•]. A local champion can: facilitate agreement on the common aims and objectives, and outcome measurements within local health; identify and mobilise relevant local resources; identify and operationalize the relevant practice changes that are needed to ensure sustainability of the programme; gather and analyse the needed data to feedback at the individual primary health care centre and community levels, leading to an adjustment of programme implementation as needed; identify and work with others in health systems to ensure that primary health care centres can access and coordinate a range of needed services and support systems; and, create a system of regular planned communication with and between primary health care centres and local communities.

### Tailoring

To ensure acceptance, local ownership and ongoing sustainability in delivery screening and brief advice programmes, all training and implementation guidelines and materials require local tailoring. Tailoring can be guided by the seven domains of the Tailored Implementation for Chronic Diseases initiative [63–65]. These are: (1) local and national guideline factors; (2) individual health care provider factors; (3) patient factors; (4) interactions between different professional groups; (5) incentives and resources; (6) capacity for organisational change; and, (7) social, political and legal factors. At the level of primary health care, tailoring should be based on the principles of co-production of health between providers and patients [66]. At the local level, tailoring should be based on the principles of integration between primary health care and local community services [67] to prevent, manage and treat heavy drinking.

### Training

Training of primary health care providers needs to cover the practical skills in undertaking screening and in delivering brief advice, in using and scoring screening instruments, and in knowing when and how to refer patients with AUD [68–71]. In addition, training should cover practice management skills, and should discuss and address providers’ attitudes, and the perceived barriers and facilitators [72–74] in implementing screening and brief advice. All aspects of training should be contextualised to local circumstances [75•].

### Adoption Mechanisms

Five adoption mechanisms have been identified to support the scale-up and implementation of prevention and treatment within health systems. As applied to screening and brief advice programmes, these include: (i) communicating the added value of primary health care-based screening and brief advice, its simplicity to deliver during a regular consultation, and its basis on the most up to date evidence of preventing and managing heavy drinking; (ii) involving respected primary health care leaders, building their skills of leadership to ensure widespread uptake of primary health care-base screening and brief advice, through identifying and advocating for large-scale change [76–78]; (iii) communicating the added value of implementing screening and brief advice programmes to both primary health care providers and to local community services [79]; (iv) identifying and advocating for appropriate and possible adjustment to local policies that influence the behaviour of primary health care providers to facilitate uptake and sustainability of screening and brief advice programmes; and, (v) bringing to the fore existing gaps in health system performance and the urgent need to prevent and manage heavy drinking as a call to motivate primary health care providers to be more active in delivering screening and brief advice programmes [80].

### Support Systems

Five support mechanisms have also been identified to support ongoing scale-up. As applied to screening and brief advice programmes, these include: (i) developing the professional capacity for scale-up amongst health care professional bodies, and commissioners and funders of health services, including insurance companies; (ii) developing, through redesign rather than additional resources the needed infrastructure for scale-up, including, for example, adjustment to electronic health records; (iii) linking implementation to regular monitoring and evaluation, with, for example, regular feedback and benchmarking of performance in undertaking screening and brief advice activity; (iv) creating intelligent systems that capture new ideas for change from implementing providers and actors, learn from collected ideas, use them to adjust the screening and brief advice programmes and their implementation. Knowledge should be shared between all implementers at primary health care and local levels through regular electronic newsletters and communications [81]; and, (v) identifying design factors that can be adjusted or implemented to ensure sustainability based on high levels of reliability and validity of new programmes, monitoring procedures to ensure that high-quality results are being achieved, and that support for structural elements, and ongoing learning systems are being implemented [82, 83].

**Table:** 

Community-based implementation strategies to enhance the volume of screening and brief advice delivered include appointing a local champion, tailoring all materials to local needs, providing training contextualised to local circumstances, communicating the added value of the programme to local communities, and providing regular feedback and benchmarking of performance.	

## Conclusions

The first trials reporting evidence for the impact of brief advice delivered in primary health care to reduce heavy drinking were published between 25 and 30 years ago [84–86]. Despite a plethora of calls to implement routine screening in primary health care and the offer of advice to screen-positive patients [87, 88, 89, 90••], there remains a large gap in delivering such programmes. There is a need to speed up the translational process so that a highly modifiable disease burden can be addressed. Such an imperative has been called for by United Nations sustainable development target 3.5, more so in times of austerity and huge pressures on health services.

The Organisation for Economic Cooperation and Development (OECD), has modelled the potential health and economic impacts of primary health care-based screening and brief advice programmes. OECD found that, were the proportion of eligible patients receiving advice and treatment for heavy drinking to be 30% of eligible patients, the prevalence of harmful use of alcohol would decrease by between 10 and 15% across OECD member countries, with reductions in the annual incidence of alcohol use disorder of between 5 and 14% [11•]. OECD noted that, although widespread implementation of such programmes can be expensive because of staff and drug costs, they bring the potential of large reductions in health care expenditures, with, in some countries, such programmes reducing costs by large margins [11•]. Primary health care-based screening and brief advice can also reduce alcohol-related diseases amongst large numbers of working age people.

The US Surgeon General’s Report on Alcohol, Drugs, and Health concluded that “supported scientific evidence indicates that substance misuse and substance use disorders can be reliably and easily identified through screening and that brief interventions work with mild severity alcohol use disorders” [90••]. The US Preventive Services Task Force recommended that “clinicians screen adults aged 18 years or older for alcohol misuse and provide persons engaged in risky or hazardous drinking with brief behavioral counseling interventions to reduce alcohol misuse” [91].

As the Phase IV WHO Collaborative Project on Identification and Management of Alcohol-related Problems in Primary Health Care, Development of Country-wide Strategies for Implementing Early Identification and Brief Intervention in Primary Health Care concluded, what is needed next are adequately controlled community-based studies to strengthen the evidence base for achieving routine implementation of screening and brief advice programmes in primary health care [58••].

## References

[1] Goldstein MG, Whitlock EP, DePue J (2004). Multiple behavioral risk factor interventions in primary care: summary of research evidence. Am J Prev Med.

[2] Mhgap Mental Health Gap Action Programme: Scaling up care for mental, neurological, and substance use disorders**.** http://www.who.int/mental_health/evidence/mhGAP/en/. 26290926

[3] Kohn R (2004). The treatment gap in mental health care. Bull World Health Organ.

[4] • Rehm J, Gmel GE, Gmel G, et al. The relationship between different dimensions of alcohol use and the burden of disease - an update. Addiction. 2017; 10.1111/add.13757. **Very comprehensive review of the health harms caused by alcohol, with a description of their dose-response relationships*****.*** 10.1111/add.13757 PMC5434904 28220587

[5] GBD 2015 Risk Factors Collaborators (2016). Global, regional and national comparative risk assessment of 79 behavioural, environmental and occupational, and metabolic risks or clusters of risks, 1990-2015: a systematic analysis for the Global Burden of Disease Study 2015. Lancet.

[6] International Agency for Research on Cancer (1988). Alcohol drinking.

[7] International Agency for Research on Cancer (2010). IARC monographs on the evaluation of carcinogenic risks to humans: alcohol consumption and ethyl carbamate, in series IARC monographs on the evaluation of carcinogenic risks to humans: alcohol consumption and ethyl carbamate, Vol. 96.

[8] Allen L, Williams J, Townsend N (2017). Socioeconomic status and non-communicable disease behavioural risk factors in low-income and lower-middle-income countries: a systematic review. Lancet Glob Health.

[9] Rehm J, Mathers C, Popova S, Thavorncharoensap M, Teerawattananon Y, Patra J (2009). Global burden of disease and injury and economic cost attributable to alcohol use and alcohol-use disorders. Lancet.

[10] Office for National Statistics: Alcohol related deaths in the United Kingdom: registered 2013. http://www.ons.gov.uk/peoplepopulationandcommunity/healthandsocialcare/causesofdeath/bulletins/alcoholrelateddeathsintheunitedkingdom/2015-02-11. Accessed 4 Dec 2016.

[11] Organisation for Economic Co-operation and Development (2015). Tackling harmful alcohol use: economics and public health policy.

[12] Rehm J, Shield KD, Rehm MX (2013). Modelling the impact of alcohol dependence on mortality burden and the effect of available treatment interventions in the European Union. Eur Neuropsychopharmacol.

[13] Rehm J, Roerecke M (2013). Reduction of drinking in problem drinkers and all-cause mortality. Alcohol Alcohol.

[14] World Health Organization: (ICD-10) http://www.who.int/substance_abuse/terminology/ICD10ClinicalDiagnosis.pdf.

[15] World Health Organization: (ICD-11) http://apps.who.int/classifications/icd11/browse/f/en#/http%3a%2f%2fid.who.int%2ficd%2fentity%2f1676588433.

[16] American Psychiatric Association (2013). Diagnostic and statistical manual of mental disorders.

[17] GBD 2015 Disease and Injury Incidence and Prevalence Collaborators (2016). Global, regional and national incidence, prevalence, and years lived with disability for 310 diseases and injuries, 1990-2015: a systematic analysis for the Global Burden of Disease Study 2015. Lancet.

[18] Whiteford HA, Ferrari AJ, Degenhardt L, Feigin V, Vos T (2015). The global burden of mental, neurological and substance use disorders: an analysis from the Global Burden of Disease Study 2010. PLoS One.

[19] GBD 2015 Mortality and Causes of Death Collaborators (2016). Global, regional and national life expectancy, all-cause mortality, and cause-specific mortality for 249 causes of death, 1980-2015: a systematic analysis for the Global Burden of Disease Study 2015. Lancet.

[20] GBD 2015 DALYs and HALE Collaborators (2016). Global, regional and national disability-adjusted life years (DALYs) for 315 diseases and injuries and healthy life expectancy (HALE), 1990-2015: a systematic analysis for the Global Burden of Disease Study 2015. Lancet.

[21] United Nations General Assembly: Political declaration of the high-level meeting of the General Assembly on the prevention and control of non-communicable diseases. http://www.un.org/en/ga/ncdmeeting2011/. Accessed 02 Jan 2017.

[22] World Health Organization (2010). Global strategy to reduce the harmful use of alcohol.

[23] United Nations. Sustainable development goal 3. https://sustainabledevelopment.un.org/sdg3. Accessed 4 Nov 2016.

[24] Rehm J (2016). How should prevalence of alcohol use disorders be assessed globally?. Int J Methods Psychiatr Res.

[25] Rehm J, Room R. Cultural specificity in measurement and in clinical responses to alcohol use disorders. Lancet. 2015; 10.1016/S0140-6736(15)00123-3.

[26] Rehm J, Marmet S, Anderson P (2013). Defining substance use disorders: do we really need more than heavy use?. Alcohol Alcohol.

[27] Rehm J, Anderson P, Gual A, et al. The tangible common denominator of substance use disorders: a reply to commentaries to Rehm et al. 2013. Alcohol and Alcoholism. 2013; 10.1093/alcalc/agt171. 10.1093/alcalc/agt171 24226811

[28] European Medicines Agency: Guideline on the development of medicinal products for the treatment of alcohol dependence. http://www.ema.europa.eu/docs/en_GB/document_library/Scientific_guideline/2010/03/WC500074898.pdf. 2010

[29] Rehm J, Room R, Monteiro M, Ezzati M (2004). Alcohol use. Comparative quantification of health risks: global and regional burden of disease attributable to selected major risk factors.

[30] Rehm J, Lachenmeier DW, Room R (2014). Why does society accept a higher risk for alcohol than for other voluntary or involuntary risks?. BMC Med.

[31] Ngo VK, Rubinstein A, Ganju V, Kanellis P, Loza N, Rabadan-Diehl C (2013). Grand challenges: integrating mental health care into the non-communicable disease agenda. PLoS Med.

[32] • Jonas DE, Garbutt JC, Brown JM, Amick HR, Brownley KA, Council CL, et al. Screening, Behavioral counseling, and referral in primary care to reduce alcohol misuse. Comparative effectiveness review No. 64. Rockville, MD: Agency for Healthcare Research and Quality; July 2012. Accessed at https://www.ncbi.nlm.nih.gov/books/NBK99199/ on 16 April 2016. **Review for the US on the impact of primary health care-based screening and brief advice in reducing heavy drinking**. 22876371

[33] Hester RK, Miller WR. Handbook of alcoholism treatment approaches. 2^nd^ ed. Boston: 1995. http://trove.nla.gov.au/version/37849636

[34] Rubinsky AD, Dawson DA, Williams EC, Kivlahan DR, Bradley KA (2013). AUDIT-C scores as a scaled marker of mean daily drinking, alcohol use disorder severity, and probability of alcohol dependence in a U.S. general population sample of drinkers. Alcohol Clin Exp Res.

[35] •• O’Donnell A, Anderson P, Newbury-Birch D, Schulte B, Schmidt C, Reimer J, et al. The impact of brief alcohol interventions in primary healthcare: a systematic review of reviews. Alcohol Alcohol. 2013; 10.1093/alcalc/agt170. **A systematic review of all reviews that convincingly demonstrates the impact of primary health care-based brief advice in reducing heavy drinking**10.1093/alcalc/agt170 PMC3865817 24232177

[36] Platt L, Melendez-Torres GJ, O’Donnell A (2016). How effective are brief interventions in reducing alcohol consumption: do the setting, practitioner group and content matter? Findings from a systematic review and metaregression analysis. BMJ Open.

[37] Bernstein J, Bernstein E, Heeren T (2010). Mechanisms of change in control group drinking in clinical trials of brief alcohol intervention: implications for bias towards the null. Drug Alcohol Rev.

[38] McCambridge J, Kypri K (2011). Can simply answering research questions change behaviour? Systematic review and meta analyses of brief alcohol intervention trials. PLoS One.

[39] Beyer FR, Kaner EFS, Hickman M, et al. Engagement with digital interventions for reducing hazardous or harmful alcohol consumption in community-dwelling populations: a systematic review: Prospero. 2015. Available from: http://www.crd.york.ac.uk/PROSPERO/display_record.asp?ID=CRD42015019790. 10.1002/14651858.CD011479.pub2 PMC6483779 28944453

[40] Kaner EFS, Beyer FR, Brown J, et al. Personalised digital interventions for reducing hazardous and harmful alcohol consumption in community-dwelling populations. Cochrane Database Syst Rev. 2015; 10.1002/14651858.CD011479/abstract. 10.1002/14651858.CD011479.pub2 PMC6483779 28944453

[41] Kaner E, Anderson P, Møller L, Galea G (2012). Health sector responses. Alcohol in the European Union: consumption, harm and policy approaches.

[42] Brown TJ, Todd A, O’Malley C (2016). Community pharmacy-delivered interventions for public health priorities: a systematic review of interventions for alcohol reduction, smoking cessation and weight management, including meta-analysis for smoking cessation. BMJ Open.

[43] Schmidt CS, Schulte B, SeoH-N KS, O’Donnell A, Kriston L, Verthein U, Reimer J (2016). Meta-analysis on the effectiveness of alcohol screening with brief interventions for patients in emergency care settings. Addiction.

[44] National Clinical Guideline Centre: The clinical management of primary hypertension in adults. 2011. https://www.nice.org.uk/guidance/cg127/evidence/cg127-hypertension-full-guideline3. Accessed 1 Dec 2016.

[45] •• Kaner EF, Dickinson HO, Beyer FR, Campbell F, Schlesinger C, Heather N, Saunders JB, Burnand B, Pienaar ED. Effectiveness of brief alcohol interventions in primary care populations. Cochrane Database Syst Rev. 2007; Issue 2. Art. No.: CD004148. doi:10.1002/14651858.CD004148.pub3. **The first fully comprehensive systematic review that demonstrates that brief interventions reduce heavy drinking by 12% on average***.* 10.1002/14651858.CD004148.pub3 17443541

[46] •• Kaner EF.S., Beyer FR, Muirhead C, Campbell F, Pienaar ED, Bertholet N, Daeppen JB, Saunders JB, Burnand B. Effectiveness of brief alcohol interventions in primary care populations. Cochrane Database Syst Rev. 2007; Issue 2. Art. No.: CD004148. doi:10.1002/14651858.CD004148.pub3. **A follow-up to the original Cochrane review that shows that when levels of alcohol consumption that trigger advice are lower, so the impact of the brief advice is lower***.*

[47] Connor JP, Haber PS, Hall WD (2016). Alcohol use disorders. Lancet.

[48] Wilson (2014). Intervention to reduce excessive alcohol consumption and improve comorbidity outcomes in hypertensive or depressed primary care patients: two parallel cluster randomized feasibility trials. Trials.

[49] Rehm J, Prieto JAA, Beier M (2016). The role of alcohol in the management of hypertension in patients in European primary health care practices – a survey in the largest European Union countries. Fam Pract.

[50] Timko C, Kong C, Vittorio L, Cucciare MA. Screening and brief intervention for unhealthy substance use in patients with chronic medical conditions: a systematic review. J Clin Nurs. 25:3131–43. 10.1111/jocn.13244. 10.1111/jocn.13244 PMC6430571 27140392

[51] Coulton S, Dale V, Deluca P, Gilvarry E, Godfrey C, Kaner E, et al. Screening for at-risk alcohol consumption in primary care: a randomised evaluation of screening approaches. Alcohol Alcohol. 2017;52:312-317. 10.1093/alcalc/agx017 28371897

[52] Anderson P, Laurant M, Kaner E, Wensing M, Grol R (2004). Engaging general practitioners in the management of hazardous and harmful alcohol consumption: results of a meta-analysis. J Stud Alcohol.

[53] Keurhorst M, van de Glind I, Bitarello do Amaral-Sabadini M, Anderson P, Kaner E, Newbury-Birch D, Braspenning J, Wensing M, Heinen M, Laurant M (2015). Determinants of successful implementation of screening and brief interventions for hazardous and harmful alcohol consumption in primary healthcare. A systematic review and meta-regression analysis. Addiction.

[54] • Anderson P, Bendsten P, Spak F, Reynolds J, Drummond C, Segura L, et al. Improving the delivery of brief interventions for heavy drinking in primary health care: outcome results of the ODHIN five-country cluster randomized factorial trial. Addiction. 2016; 10.1111/add.13476. **A systematic review that demonstrates the importance of a range of factors, including training and support, in increasing provider behaviour in delivering screening and brief advice programmes**.

[55] • Anderson, P., Gual, T., Coulton, S., Kaner, E., Bendsten, P., Kłoda, K., et al. Improving the delivery of brief interventions for heavy drinking in primary health care: nine month outcomes of the ODHIN five-country cluster randomized factorial trial. Ann Fam Pract. 2017;15:335-340. **A five-country study from Europe that demonstrates the lasting impact of relatively brief training of providers in increasing their screening and brief advice activity***.*

[56] Anderson P, Kaner E, Wutzke S, Funk M, Heather N, Wensing M (2004). Attitudes and managing alcohol problems in general practice: an interaction analysis based on findings from a WHO collaborative study. Alcohol Alcohol.

[57] O’Donnell A, Haighton C, Chappel D, Shevills C, Kaner E. Impact of financial incentives on delivery of screening and brief alcohol interventions in primary care: a mixed methods investigation. BMC Fam Pract. 2016;17:165. 10.1186/s12875-016-0561-5 PMC5124277 27887577

[58] •• Heather N. editor. WHO Collaborative Project on Identification and Management of Alcohol-related Problems in Primary Health Care – Report to the World Health Organisation on Phase IV: Development of Country-wide Strategies for Implementing Early Identification and Brief Intervention in Primary Health Care. Geneva: World Health Organisation, Department of Mental Health and Substance Abuse: 2006. http://www.who.int/substance_abuse/publications/identification_management_alcoholproblems_phaseiv.pdf. **One of the major studies in the field that attempted to disseminate uptake of screening and brief advice in primary health care country wide. Concluded that, to be successful, primary health care activities need to be embedded within broader community and municipal action***.*

[59] Babor T, Del Boca F, Bray JW (2017). Screening, brief intervention and referral to treatment: implications of SAMHSA’s SBIRT initiative for substance abuse policy and practice. Addiction.

[60] Vendetti J, Gmyrek A, Damon D, Singh M, McRee B, Del Boca F (2017). Screening, brief intervention and referral to treatment (SBIRT): implementation barriers, facilitators and model migration. Addiction.

[61] Singh M, Gmyrek A, Hernandez A, Damon D, Hayashi S (2017). Sustaining screening, brief intervention and referral to treatment (SBIRT) services in health-care settings. Addiction.

[62] Barker PM, Reid A, Schall MW (2016). A framework for scaling up health interventions: lessons from large-scale improvement initiatives in Africa. Implement Sci.

[63] Wensing M, Oxman A, Baker R, Godycki-Cwirko M, Flottorp S (2011). Tailored implementation for chronic diseases (TICD): a project protocol. Implement Sci.

[64] Wensing M, Huntink E, van Lieshout J, Godycki-Cwirko M, Kowalczyk A (2014). Tailored implementation of evidence-based practice for patients with chronic diseases. PLoS One.

[65] Flottorp SA, Oxman AD, Krause J, Musila NR, Wensing M (2013). A checklist for identifying determinants of practice: a systematic review and synthesis of frameworks and taxonomies of factors that prevent or enable improvements in healthcare professional practice. Implement Sci.

[66] Dietz WH, Solomon LS, Pronk N, Ziegenhorn SK, Standish M, Longjohn MM, Fukuzawa DD, Eneli IU, Loy L, Muth ND, Sanchez EJ, Bogard J, Bradley DW (2015). An integrated framework for the prevention and treatment of obesity and its related chronic diseases. Health Aff.

[67] Palumbo R (2016). Contextualizing co-production of health care: a systematic literature review. Int J Public Sect Manag.

[68] Babor TF, Higgins-Biddle JC, Higgins PS, Gassman RA, Gould BE (2004). Training medical providers to conduct alcohol screening and brief interventions. Subst Abus.

[69] Fleming MF (1997). Strategies to increase alcohol screening in health care settings. Alcohol Res Health.

[70] Davis DA, Thomson MA, Oxman AD, Haynes RB (1995). Changing physician performance: a systematic review of the effect of continuing medical education strategies. JAMA.

[71] Fleming MF (2004). Screening and brief intervention in primary care settings. Alcohol Res Health.

[72] Garzonis K, Mann E, Wyrzykowska A, Kanellakis P (2015). Improving patient outcomes: effectively training healthcare staff in psychological practice skills: a mixed systematic literature review. Eur J Psychol.

[73] Nilsen P, Wåhlin S, Heather N (2011). Implementing brief interventions in health care: lessons learned from the Swedish risk drinking project. Int J Environ Res Public Health.

[74] Patterson Silver Wolf DA (2015). Factors influencing the implementation of a brief alcohol screening and educational intervention in social settings not specializing in addiction services. Soc Work Health Care.

[75] • May CR, Johnson M, Finch T. Implementation, context and complexity. Implementation Science. 2016; 10.1186/s13012-016-0506-3. **Stresses the importance of tailoring all programmes to fit local circumstances*****.*** 10.1186/s13012-016-0506-3 PMC5069794 27756414

[76] McCannon CJ, Perla RJ (2009). Learning networks for sustainable, large-scale improvement. Jt Comm J Qual Patient Saf.

[77] Reinertsen JL, Bisognano M, Pugh MD. Seven leadership leverage points for organization-level improvement in health care. 2nd ed. (IHI innovation series white paper) ed. Cambridge: Institute for Healthcare Improvement; 2008.

[78] Swenson S, Pugh M, McMullan C, Kabcenell A (2013). Book high impact leadership: improve care, improve the health of populations, and reduce costs (IHI white paper).

[79] Rogers E (2003). Diffusion of innovations.

[80] McCannon CJ, Schall MW, Perla RJ (2008). Planning for scale: a guide for designing large-scale improvement initiatives (IHI innovation series white paper).

[81] Schouten LM, Hulscher ME, van Everdingen JJ, Huijsman R, Grol RP (2008). Evidence for the impact of quality improvement collaboratives: systematic review. Br Med J.

[82] NHS Modernisation Agency (2002). Improvement leaders guide to sustainability and spread.

[83] Mate K, Sifrim Z, Provost L (2011). Sustainability of improvement interventions (IHI 90-day R&D project final summary report).

[84] Wallace P, Cutler S, Haines A (1988). Randomised controlled trial of general practitioner intervention in patients with excessive alcohol consumption. Br Med J.

[85] Scott E, Anderson P (1990). Randomized controlled trial of general practitioner intervention in women with excessive alcohol consumption. Drug and Alcohol Review.

[86] Anderson P, Scott E (1992). The effect of general practitioners’ advice to heavy drinking men. Br J Addict.

[87] National Institute for Health and Clinical Excellence: Alcohol-use disorders - preventing the development of hazardous and harmful drinking. 2010. https://www.nice.org.uk/guidance/ph24.

[88] Dua T, Barbui C, Clark N, Fleischmann A, Poznyak V, van Ommeren M (2011). Evidence based guidelines for mental, neurological and substance use disorders in low- and middle-income countries: summary of WHO recommendations. PLoS Med.

[89] Health Scotland: Alcohol Advice 2012/2013. 2013. https://isdscotland.scot.nhs.uk/Health-Topics/Drugs-and-Alcohol-Misuse/Publications/2013-06-25/2013-06-25-ABI-Report.pdf?89017885924.

[90] •• U.S. Department of Health and Human Services (HHS), Office of the Surgeon General, Facing Addiction in America: The Surgeon General’s Report on Alcohol, Drugs, and Health. Washington, DC: HHS, November 2016. **A major US report that stressed the importance of primary health care-based screening and brief advice programmes, within the broader context of the need for treatment for alcohol use and other substance disorders***.* 28252892

[91] US Preventive Services Task Force: Alcohol misuse: screening and behavioral counseling interventions in primary care. Release date: 2013. https://www.uspreventiveservicestaskforce.org/Page/Document/UpdateSummaryFinal/alcohol-misuse-screening-and-behavioral-counseling-interventions-in-primary-care.

